# Effectiveness of savoring interventions: A systematic review and meta‐analysis of randomized controlled trials

**DOI:** 10.1111/aphw.70134

**Published:** 2026-03-08

**Authors:** Pei‐Hsin Chen, Heng‐Hsin Tung, Yu‐Chen Wu, Joanna Sie

**Affiliations:** ^1^ College of Nursing National Yang Ming Chiao Tung University Taipei City Taiwan; ^2^ Department of Nursing Taichung Veterans General Hospital Taichung City Taiwan; ^3^ Taiwan Association of Nursing Practitioner New Taipei City Taiwan; ^4^ Graduate Institute of Clinical Nursing, College of Medicine National Chung Hsing University Taichung Taiwan; ^5^ Department of Nursing National Taiwan University Hospital Taipei City Taiwan

**Keywords:** positive emotions, randomized controlled trials, savoring intervention, three‐level meta‐analysis, well‐being

## Abstract

Savoring, a positive psychology intervention, has gained growing attention for its potential to enhance positive emotions and well‐being while alleviating negative emotional symptoms such as depression and anxiety. This systematic review and meta‐analysis synthesized findings from randomized controlled trials (RCTs) evaluating the efficacy of savoring interventions on emotional outcomes. Twenty independent RCTs with 4805 participants were included, yielding 45 effect sizes. A three‐level random‐effects model with robust variance estimation (CR2) was employed to account for statistical dependence among multiple effects within studies. Risk of bias was assessed using the Cochrane Risk of Bias 2.0 tool. All analyses were conducted in R version 4.5.1 using the metafor (v4.8.0) and clubSandwich (v0.6.1) packages, with statistical significance set at *α* = .05 (two‐tailed). The overall pooled effect was significant and of moderate magnitude (*g* = 0.51, 95% CI [0.26, 0.77], *p* < .001). For descriptive purposes, category‐specific pooled estimates were observed for negative emotional symptoms (*g* = 0.61, 95% CI [0.31, 0.91], *p* < .001), negative emotional states (*g* = 0.33, 95% CI [−0.01, 0.68], *p* = .056), and positive psychological states (*g* = 0.50, 95% CI [0.24, 0.75], *p* < .001); outcome category did not significantly moderate intervention effects. Moderator analyses indicated no statistically significant differences across delivery formats, cultural contexts, or risk of bias levels; control group type did not reach statistical significance as a moderator (Δ*g* = −0.47, *p* = .068), although point estimates were larger for passive than active controls. Publication bias analyses (Egger's regression, PET‐PEESE, trim‐and‐fill, and selection models) provided convergent evidence that the observed effects were unlikely to be driven by selective reporting. Savoring interventions demonstrate consistent and meaningful benefits for enhancing positive psychological outcomes and reducing negative emotional symptoms across diverse populations and delivery modes. The findings underscore savoring's potential as a scalable and culturally adaptable approach to promoting emotional well‐being.

## INTRODUCTION

Mental health is a fundamental human right and an essential component of overall health and well‐being (WHO, 2022). However, in modern society, challenges such as work‐related stress, social media comparisons, the impact of the pandemic, and economic instability have contributed to a continued rise in the prevalence of mental health problems. According to the World Health Organization (2022), the prevalence of anxiety and depression increased by 25% during the first year following the COVID‐19 outbreak, underscoring the urgent need to strengthen strategies for promoting mental health.

Among the various protective factors, positive emotions are regarded as a central pathway to mental health. They not only help individuals discover happiness and meaning in life and are strongly associated with subjective well‐being but also hold the potential to prevent the onset of mental disorders (Fredrickson, 2001; Silton et al., 2020; Yang, 2020). In recent years, the field of psychology has increasingly emphasized how intervention strategies can effectively cultivate and sustain positive emotions. Among these, *savoring*, defined as the conscious process of attending to, extending, and amplifying positive experiences, has been identified as a key mechanism for enhancing positive emotions and well‐being (Bryant & Veroff, 2007).

Positive emotions are an important pathway to promoting mental health, as they help individuals discover joy and meaning in life and hold the potential to prevent psychological disorders (Silton et al., 2020). They are also strongly associated with subjective well‐being (Fredrickson, 2001; Yang, 2020). Increasing empirical evidence indicates that interventions targeting the regulation of positive emotions can yield significant benefits. For example, Niemann et al. (2023) found that training in positive emotions effectively reduced depression and anxiety while enhancing optimism. Earlier landmark trials also support this view: Craske et al. (2019) demonstrated that positive affect treatment (PAT) was superior to negative emotion‐focused therapies in enhancing positive affect, alleviating emotional symptoms, and reducing suicidal ideation. Similarly, Taylor et al. (2020) showed that the amplification of positivity (AMP) intervention strengthened positive emotions and social connectedness, helping to alleviate social disconnection among individuals with anxiety and depression. In clinical practice, positive psychology interventions have been recognized as a feasible and effective approach to promoting mental health (Huffman et al., 2022). Since the publication of *Savoring: A New Model of Positive Experience* (Bryant & Veroff, 2007), research on savoring has grown rapidly and has gradually become a central concept in positive psychology (Bryant, 2021).

Savoring refers to the process by which individuals consciously notice, deeply experience, prolong, and amplify positive experiences. This process enhances individuals' sense of well‐being and their ability to regulate positive emotions (Bryant & Veroff, 2007). Sato et al. (2018) found that savoring mediated the relationship between engagement in positive activities and the generation of positive emotions. Specifically, savoring enhanced individuals' positive emotional experiences by deepening their awareness of and attention to pleasurable moments, which in turn increased their reported positive affect. Moreover, savoring also moderates the relationship between health and life satisfaction among older adults, such that those with higher savoring ability maintain greater life satisfaction even in the context of poor physical health (Smith & Bryant, 2016). Savoring has been shown to enhance and sustain positive emotional experiences over time, producing lasting emotional and neural effects (Wilson & MacNamara, 2020). This intervention is simple and easy to learn and yields immediate results, making it widely applicable to both clinical and nonclinical populations.

Recently, savoring has garnered attention as an important positive psychological intervention (PPI) strategy. Related studies have highlighted its positive effects on alleviating depression, reducing anxiety, and enhancing subjective well‐being (Bryant, 2021; Cullen et al., 2024; Zheng et al., 2025). Savoring interventions during the COVID‐19 pandemic demonstrated significantly enhanced subjective well‐being, which made them a practical and feasible strategy for promoting mental health (Villani et al., 2023). Studies revealed that savoring interventions can be applied successfully across various contexts, including stress regulation (Nicolson et al., 2020), enhancement of positive emotions (Klibert et al., 2022), alleviation of negative emotions (Contractor et al., 2020), promotion of psychological well‐being in older adults (Smith et al., 2020), chronic pain relief (Finan et al., 2023), and reductions in depression (Bastiaansen et al., 2022; Selva et al., 2012; Serrano et al., 2004) and anxiety symptoms (LaFreniere & Newman, 2023a). Savoring strategies may include three behavioral dimensions (sharing with others, full attention, and behavioral expression) and seven cognitive dimensions (downward comparison, heightened perception, memory building, self‐motivation, appreciation of transience, counting blessings, and dampening avoidance), among others. These can prolong and amplify positive emotions, enhance well‐being, and reduce negative affect (Bryant & Veroff, 2007).

Increasingly, studies have supported the effectiveness of regular savoring practices in improving overall psychological health (Bastiaansen et al., 2022; LaFreniere & Newman, 2023a; Villani et al., 2023; Yu et al., 2020). However, most have been small‐scale case studies or short‐term interventions and lack a comprehensive evaluation of overall efficacy. Furthermore, among studies with lower methodological rigor, notable differences in intervention content, delivery methods, and outcome measures increase the difficulty of comparing findings across studies. To date, few systematic reviews and meta‐analyses have been conducted on this topic. Zheng et al. (2025) performed a meta‐analysis focused exclusively on student populations, which limited the generalizability of their findings. Furthermore, Cullen et al. (2024) conducted a systematic review that targeted adult clinical populations; however, because of considerable heterogeneity in intervention types and outcome assessments, a meta‐analysis was not deemed feasible by its authors.

Therefore, this study aimed to conduct a comprehensive systematic review and meta‐analysis across diverse populations, contexts, and cultures. In addition, it synthesized existing randomized controlled trials (RCTs) to evaluate the effectiveness of savoring interventions in promoting positive emotions, reducing negative emotions, and enhancing well‐being. Additionally, we examined six prespecified moderators: emotional outcome category (negative symptoms, negative states, positive states), cultural context (Eastern vs. Western), intervention format, duration, control type, and risk of bias. Notably, although interventions such as gratitude and best possible self (BPS) include elements related to the savoring process, previous studies have conducted meta‐analyses on their impact on well‐being (Carrillo et al., 2019; Diniz et al., 2023). To maintain conceptual clarity and a consistent operational definition, this study focused on interventions that explicitly targeted savoring processes and excluded other positive psychology interventions that are conceptually distinct, such as gratitude, BPS, and acts of kindness interventions. Although all three types of interventions can enhance positive emotions, their core mechanisms differ conceptually and procedurally from savoring. Gratitude interventions emphasize interpersonal appreciation and thankfulness (Salces‐Cubero et al., 2019); BPS interventions focus on future‐oriented visualization and goal realization (King, 2001); and acts of kindness interventions center on outward prosocial behaviors that promote social connectedness and self‐worth (Lyubomirsky et al., 2005), rather than deliberate engagement with or prolongation of current positive emotions (Bryant & Veroff, 2007). Therefore, interventions that do not meet the operational definition of savoring, namely, the conscious regulation, amplification, or prolongation of positive emotional experiences, were excluded. This selection criterion was designed to maintain construct purity, prevent conceptual confounding, and ensure a clear and consistent research focus.

To contextualize the current review within the existing literature, we systematically compared our synthesis with prior work on savoring interventions, including Zheng et al. (2025) and the related systematic review by Cullen et al. (2024). The comparison covered key methodological and analytical features, including PICO frameworks, search periods, number of included studies, summary effect sizes, heterogeneity estimates, and reported moderators. To assess the degree of study‐level redundancy between our review and Zheng et al. (2025), we used the GROOVE tool (Graphical Representation of Overlap for OVErviews). The GROOVE analysis yielded a corrected covered area (CCA) of 21.4%, which, according to established thresholds (Bracchiglione et al., 2022), indicates a very high degree of overlap. This suggests substantial evidence redundancy while also highlighting the value of an updated synthesis given newly published trials and methodological refinements. A full comparison is presented in Table S1, offering a transparent overview of how the present meta‐analysis expands upon and extends prior findings in this domain.

## METHODS

### Search strategy

This systematic review and meta‐analysis were conducted in accordance with the Preferred Reporting Items for Systematic Reviews and Meta‐Analyses (PRISMA) guidelines (Moher et al., 2009) (Table S2). The review protocol was preregistered on INPLASY (registration number: NPLASY202530114).

Two independent reviewers (P.‐H. Chen and J. Sie) conducted comprehensive electronic searches across PubMed, PsycINFO, Cochrane Library, CINAHL, MEDLINE, and Google Scholar, from inception till March 6, 2025. The following combination of keywords and Boolean operators were used: (“savoring” OR “savouring” OR “savoring intervention” OR “savouring intervention” OR “savoring the moment” OR “savouring the moment” OR “three good thing*” OR “self‐congratulations” OR “memory building” OR “positive emotion regulation” OR “positive life review”) AND (“randomized controlled trial*”) AND (“positive emotion*” OR “positive affect*” OR “well‐being” OR “happiness” OR “spiritual well‐being” OR “PANAS” OR “depression” OR “anxiety”). Additional records were identified by screening the reference lists of the eligible articles and relevant review papers. The detailed search strategy for this systematic review and meta‐analysis is provided in the Supporting Information (Table S3).

### Inclusion and exclusion criteria

To ensure conceptual clarity and distinguish savoring‐based interventions from other PPIs, we adopted specific inclusion and exclusion criteria guided by the Population, Intervention, Comparison, Outcome (PICO) framework. Population (P): Human participants of any age or health condition were included. Intervention (I): Savoring interventions were defined as programs explicitly designed to enhance savoring, characterized by the intentional focus on, amplification of, and prolonged engagement with positive emotional experiences across past, present, or future timeframes (Bryant, 2021; Rosen & LaFreniere, 2023). Comparison (C): Any type of control group was eligible, including no intervention, treatment as usual, placebo, or active comparator. Outcomes (O): Eligible outcomes were limited to emotional or mental health variables, specifically positive affect, negative affect, well‐being, happiness, depression, or anxiety. These outcomes had to be assessed both preintervention and postintervention and provide sufficient data (e.g. means and standard deviations) to compute standardized effect sizes. Only RCTs published in English or Chinese were eligible. Exclusion criteria encompassed non‐RCT designs, review articles, protocols, case reports, qualitative studies, conference abstracts, and studies with unusable or incomplete outcome data.

In addition, interventions primarily targeting gratitude (e.g. gratitude letters), kindness (e.g. helping behaviors), or the BPS were excluded. Although these interventions may also enhance positive emotions, they do not conform to our operational definition of savoring adopted in this study, which emphasizes the deliberate regulation and amplification of positive emotions that are occurring or directly experienced. The theoretical foundation of the BPS intervention originated from Pennebaker's expressive writing paradigm and aims to promote psychological health through writing about future‐oriented goals and ideal self‐realization (King, 2001). In contrast, savoring focuses on the awareness, maintenance, and emotional regulation of actual positive experiences (Bryant & Veroff, 2007), and thus, the two rely on distinct mechanisms. Furthermore, the meta‐analyses by Carrillo et al. (2019) and Diniz et al. (2023) indicated that BPS fosters positive emotions through envisioning one's ideal future self, whereas gratitude interventions enhance well‐being by appreciating life's blessings. Their underlying mechanisms are conceptually distinct from the core savoring processes examined in this study. Multicomponent interventions were also excluded if the effects of savoring were not independently analyzed.

### Methodological quality appraisal

Two reviewers (P. H. Chen and J. Sie) independently evaluated the methodological quality of the included RCTs via the Cochrane Collaboration's Risk of Bias tool. This tool assessed seven domains: random sequence generation and allocation concealment (selection bias), blinding of participants and personnel (performance bias), blinding of outcome assessment (detection bias), incomplete outcome data (attrition bias), selective reporting (reporting bias), and other potential sources of bias.

For the domain of *blinding of outcome assessment*, we defined “outcome assessors not blinded” as either (a) outcome evaluations conducted by assessors who were aware of participants' group allocation or (b) outcomes measured through self‐report questionnaires completed by participants without interviewer involvement. Each domain was rated as having a low, some concern, or high risk of bias. Any discrepancies or uncertainties were resolved through discussions with a third reviewer (H. H. Tung) (Higgins & Thomas, 2024).

### Outcomes

The outcomes were changes in positive and negative affect scale scores from pre– to post–savoring intervention. Positive affect included measures of positive emotions and well‐being, whereas negative affect encompassed negative emotional syndromes or disorders, such as depression and anxiety. To assess both positive and negative affect, studies commonly used the *Positive and Negative Affect Schedule* (PANAS). Additional validated instruments for positive affect and well‐being included the *Life Satisfaction Index* (LSI), *Subjective Happiness Scale* (SHS), and *McGill Quality of Life Questionnaire* (MQOL). To evaluate negative emotional syndromes or disorders, such as depression and anxiety, the studies employed the Center for Epidemiological Studies Depression Scale (CES‐D), Geriatric Depression Scale (GDS), Patient Health Questionnaire‐9 (PHQ‐9), Hospital Anxiety and Depression Scale (HADS), Beck Depression Inventory‐II (BDI‐II), World Health Organization‐Five Well‐Being Index (WHO‐5), Penn State Worry Questionnaire (PSWQ), and Inventory for Depressive Symptomatology‐Self‐Report (IDS‐SR).

### Study selection, data extraction, and outcome mapping

Eligible studies were RCTs of savoring interventions with a comparator arm. Two reviewers (P. H. Chen and J. Sie) independently extracted study characteristics and outcome data (means, standard deviations, sample sizes, test statistics, and measurement occasions). For transparency, effect sizes were first computed within the five outcome domains commonly reported in the literature (anxiety, depression, negative affect, positive affect, well‐being). For meta‐analytic modeling and reporting, these domains were then mapped into three a priori categories: negative emotional disorders (integrating depression and anxiety outcomes), negative emotional states, and positive psychological states. Effect directions were harmonized so that positive values consistently indicate the benefit of the savoring intervention (negative outcomes were sign‐reversed). Multiarm trials and multiple outcomes per study were retained; statistical dependence was addressed at the modeling stage.

### Data analysis and effect size computation (Campbell/Wilson calculator)

All effects were expressed as Hedges' *g* (bias‐corrected standardized mean difference), computed using the Campbell Collaboration Practical Meta‐Analysis Effect Size Calculator (Wilson, 2023), which implements the formulas in Hedges and Olkin (1985) and Morris (2008).

(A) When pretest and posttest means (M), standard deviations (*SD*), and group sizes (n_E, n_C) were available, we used the “Means, *SD*s with Pretest” approach corresponding to Morris's d_ppc2 (difference‐in‐differences standardized by the pooled pretest *SD*). The small‐sample correction was *g* = *J* × *d*, with *J* = 1–3/[4(n_E + n_C − 2) − 1]. The pre–post correlation (*r*) was obtained from paired *t* when reported; otherwise, *r* = 0.50 was assumed. Note that for d_ppc2, the point estimate *g* does not depend on *r*, whereas the standard error and sampling variance do.

(B) When only the results of a two‐sample comparison were available (*t* or *p* with degrees of freedom), and the statistic referred to an independent‐samples test rather than ANCOVA/regression, we used the calculator's “*t*‐tests with unequal *N*s” option and the exact relation *d* = *t* × $\sqrt{\left(1/n\_E+1/n\_C\right)}$ (or *t* = $\sqrt{F}$ when the numerator *df* = 1), followed by the same Hedges' *J* correction to obtain *g*. If only a *p*‐value was reported, *t* was recovered from *p* and the reported *df*. Sampling variances were computed using the calculator's unbiased expressions for each route.

### Three‐level meta‐analysis

Given that multiple effect sizes were extracted from individual studies (*k* = 45 effect sizes from *K* = 20 studies), we employed three‐level random‐effects meta‐analytic models to account for statistical dependence (Assink & Wibbelink, 2016; Cheung, 2014; Van den Noortgate et al., 2013). The three‐level structure partitioned variance into sampling variance (Level 1), within‐study heterogeneity (Level 2), and between‐study heterogeneity (Level 3). All models used restricted maximum likelihood (REML) in the metafor package (Viechtbauer, 2010). Cluster‐robust variance estimation with CR2 small‐sample corrections (Pustejovsky & Tipton, 2022) was applied for all hypothesis tests and confidence intervals.

### Heterogeneity assessment

We first assessed statistical heterogeneity by examining the overall variance among effect sizes using Cochran's *Q*‐test. Given that heterogeneity was expected, we then partitioned this total variance into Level 3 (between‐study *σ* ^2^) and Level 2 (within‐study *σ* ^2^) components. The *I* ^2^ statistic was subsequently used to quantify the percentage of variance attributable to each of these sources. To statistically justify this three‐level approach over a standard two‐level model, likelihood ratio tests (LRTs) were employed to determine if the variance components at both levels were significantly greater than zero.

### Moderator analysis

We examined prespecified moderators: outcome category (negative disorder, negative states, positive states), cultural context (Eastern vs. Western), intervention format, duration, control type, and risk of bias. Omnibus tests used cluster‐robust Wald tests with Hotelling–Zhang adjustments (Tipton & Pustejovsky, 2015) to control Type I error.

### Publication bias assessment

We used methods adapted for three‐level structures with dependent effect sizes: (1) contour‐enhanced funnel plots for visual inspection, with contours centered at the null effects (Peters et al., 2008); (2) RVE‐adjusted Egger's regression test (Rodgers & Pustejovsky, 2021); (3) PET‐PEESE meta‐regression for small‐study effects (Stanley & Doucouliagos, 2014); and (4) trim‐and‐fill analysis on study‐aggregated effects (Duval & Tweedie, 2000). Given substantial heterogeneity (*I* ^2^ > 80%), we interpreted all indicators cautiously and prioritized convergent evidence.

## RESULTS

### Study identification and selection

**FIGURE 1:**
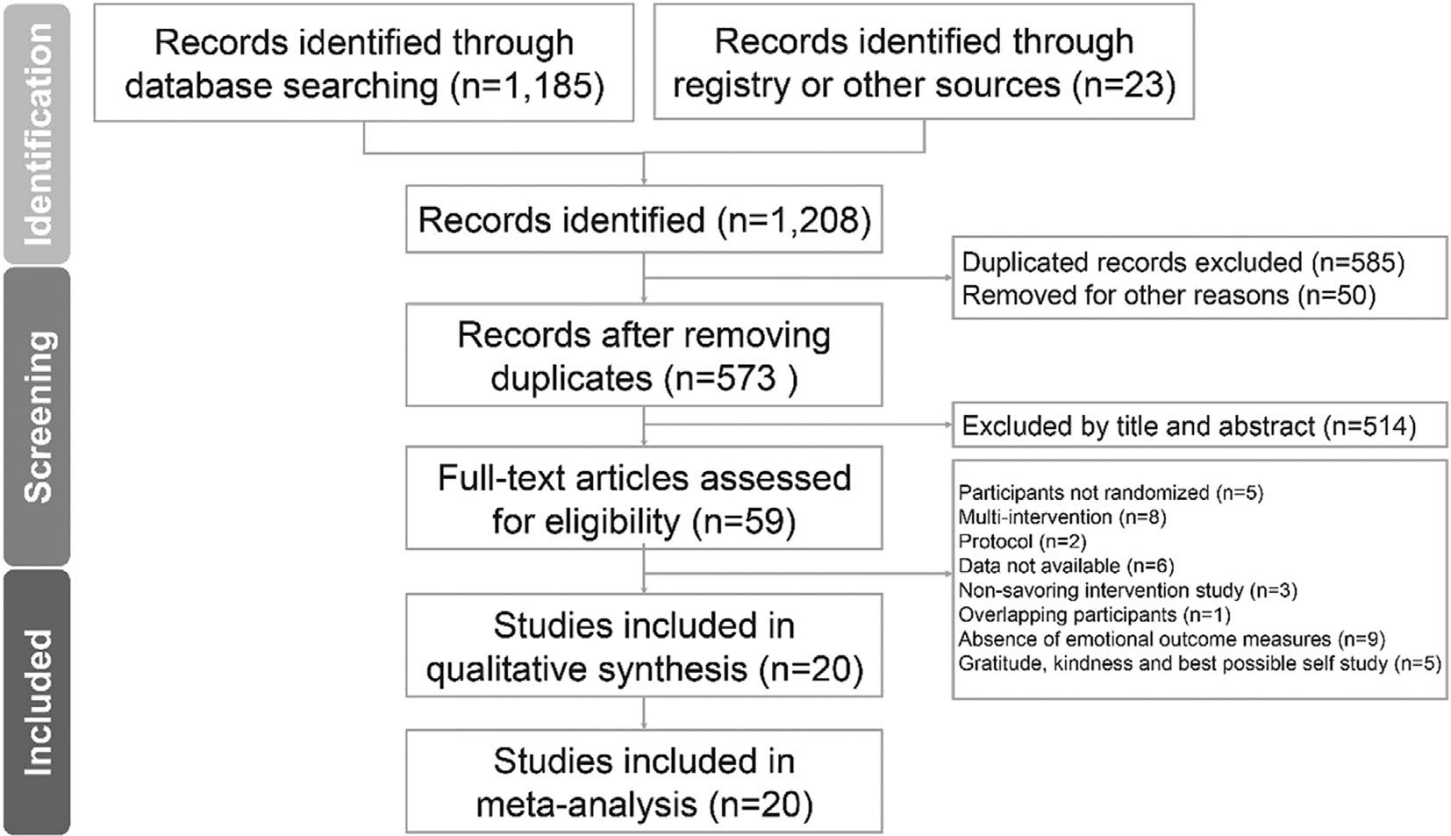
PRISMA 2020 flowchart for the current meta‐analysis.

Figure 1 presents the PRISMA flowchart for updated systematic reviews, which includes searches of databases, registers, and other sources.

A total of 1185 titles were retrieved from the databases, and an additional 23 records were identified through existing meta‐analyses. After duplicates were removed, 573 records were screened, of which 514 were excluded after the abstracts were reviewed. Finally, 59 articles were identified as potentially eligible studies, of which 39 did not meet the inclusion criteria. Reasons for exclusion included the use of multicomponent intervention (*n* = 8) (Garland et al., 2022; Garland et al., 2024; Ho et al., 2016a, 2016b; Kumar et al., 2024; Moskowitz et al., 2019; Ong et al., 2022; Seligman et al., 2005); absence of emotional outcome measures (*n* = 9) (Borelli et al., 2020; Borelli, Kazmierski, et al., 2023; Borelli et al., 2024; Cheng et al., 2023; Irvin et al., 2022; LaFreniere & Newman, 2023b; Palmer et al., 2024; Smiley et al., 2024; Straszewski & Siegel, 2018); overlapping participants (*n* = 1) (LaFreniere & Newman, 2024); non‐RCTs (*n* = 5) (Cline et al., 2022; Smith & Hanni, 2019; Smith & Hollinger‐Smith, 2015; Tighe et al., 2022; Villani et al., 2023); protocols (*n* = 2) (Pancini et al., 2022, 2023); articles not related to savoring interventions (*n* = 3) (Ho et al., 2024; Lyubomirsky et al., 2005; Passmore & Howell, 2014); focused on gratitude, kindness, and BPS interventions (*n* = 5) (Datu et al., 2022; Deng et al., 2016; Nicolson et al., 2020; Ouweneel et al., 2014; Sheldon & Lyubomirsky, 2006); and data were not available (*n* = 6) (Borelli, Kerr, et al., 2023; Frein & Ponsler, 2013; Quoidbach et al., 2009; Rosen & LaFreniere, 2023; Smith & Bryant, 2019; Zehner et al., 2023). Details and reasons for excluded articles are listed in Table S4. Finally, 20 RCTs met the inclusion criteria. Among them, 14 studies measured negative emotional symptoms (Ando et al., 2010; Bastiaansen et al., 2022; Contractor et al., 2020; Fuju et al., 2022; Gold et al., 2023; Goncalves et al., 2009; Hurley & Kwon, 2011; Kwan et al., 2019; LaFreniere & Newman, 2023a; Li et al., 2021; McMakin et al., 2011; Selva et al., 2012; Serrano et al., 2004; Yu et al., 2020), eight studies measured negative emotional states (Contractor et al., 2020; Finan et al., 2023; Hurley & Kwon, 2011; Li et al., 2021; McMakin et al., 2011; Mian & Earleywine, 2023; Yu et al., 2020; Zhang et al., 2023), and 18 studies assessed positive psychological states (Bastiaansen et al., 2022; Bryant et al., 2005; Contractor et al., 2020; Finan et al., 2023; Fuju et al., 2022; Gold et al., 2023; Goncalves et al., 2009; Hurley & Kwon, 2011; Kwan et al., 2019; Li et al., 2021; LaFreniere & Newman, 2023a; McMakin et al., 2011; Mian & Earleywine, 2023; Klibert et al., 2022; Selva et al., 2012; Serrano et al., 2004; Spillane et al., 2023; Yu et al., 2020; Zheng et al., 2025). Details of data extraction from the included RCTs are summarized in Table S5.

### Study characteristics and methodological quality of the included studies

The meta‐analysis included 20 independent RCTs with 4805 participants, yielding 45 effect sizes. The median intervention duration was 14 days (IQR: 7–28 days). Studies were distributed across delivery formats as follows: individual (11 studies, 55%), online computer‐based (4 studies, 20%), and smartphone application (5 studies, 25%). Control conditions comprised passive controls (8 studies, 40%) and active controls (12 studies, 60%). The cultural classification is as follows: Western cultures (14 studies, 70%) and Eastern cultures (6 studies, 30%) (Table 1).

**TABLE 1:** Characteristics of studies included in meta‐analysis.

Study	Country	Population	Culture	Intervention	Duration (days)	Control	Arms	RoB
Serrano et al., 2004	Spain	Older adults	Western	Individual	28	Passive	2	Low
Bryant et al., 2005	USA	Undergraduate students	Western	Individual	7	Active	1	Some
Goncalves et al., 2009	Spain	Adult daycare center older women	Western	Individual	28	Active	2	High
Ando, 2010	Japan	Terminal ill cancer patients	Eastern	Individual	7	Passive	1	High
McMakin et al., 2011	USA	University students	Western	Individual	4	Active	3	Some
Hurley & Kwon 2011	USA	University students	Western	Individual	14	Passive	3	High
Selva et al., 2012	Spain	Older adults	Western	Individual	28	Active	2	Low
Kwan et al., 2019	Hong Kong	Terminal ill cancer patients	Eastern	Individual	7	Active	3	Low
Yu et al., 2020	China	University students	Eastern	Online	21	Passive	3	High
Contractor et al., 2020	USA	University students	Western	Individual	1	Active	3	Some
Li et al., 2021	China	Adults	Eastern	Online	28	Active	5	Some
Klibert et al., 2022	USA	University students	Western	Individual	1	Active	1	Some
Bastiaansen et al., 2022	Netherlands	Adults	Western	Smartphone	28	Passive	1	Some
Fuju et al., 2022	Japan	Adults	Eastern	Individual	28	Passive	2	Some
Finan et al., 2023	USA	Adults	Western	Online	14	Active	2	Some
Gold et al., 2023	USA	Adults	Western	Smartphone	21	Passive	3	Low
LaFreniere & Newman 2023a	USA	University students	Western	Smartphone	7	Active	3	Some
Mian & Earleywine, 2023	USA	Undergraduate students	Western	Online	7	Active	2	High
Spillane et al., 2023	USA	Adults	Western	Smartphone	14	Active	1	High
Zhang et al., 2023	China	College students	Eastern	Smartphone	14	Passive	2	Some

*Note*: Studies are listed in chronological order by publication year. Culture classified as Eastern (China, Japan, Hong Kong, Pakistan) or Western (USA, Spain, Netherlands); control types: passive (no intervention, waitlist, usual care) or active (alternative intervention with similar time/attention); RoB = Risk of Bias rating based on Cochrane RoB 2.0 tool. The “Individual” delivery format category includes traditional face‐to‐face psychotherapy sessions as well as structured, nondigital tasks completed individually, such as guided writing exercises.

**FIGURE 2:**
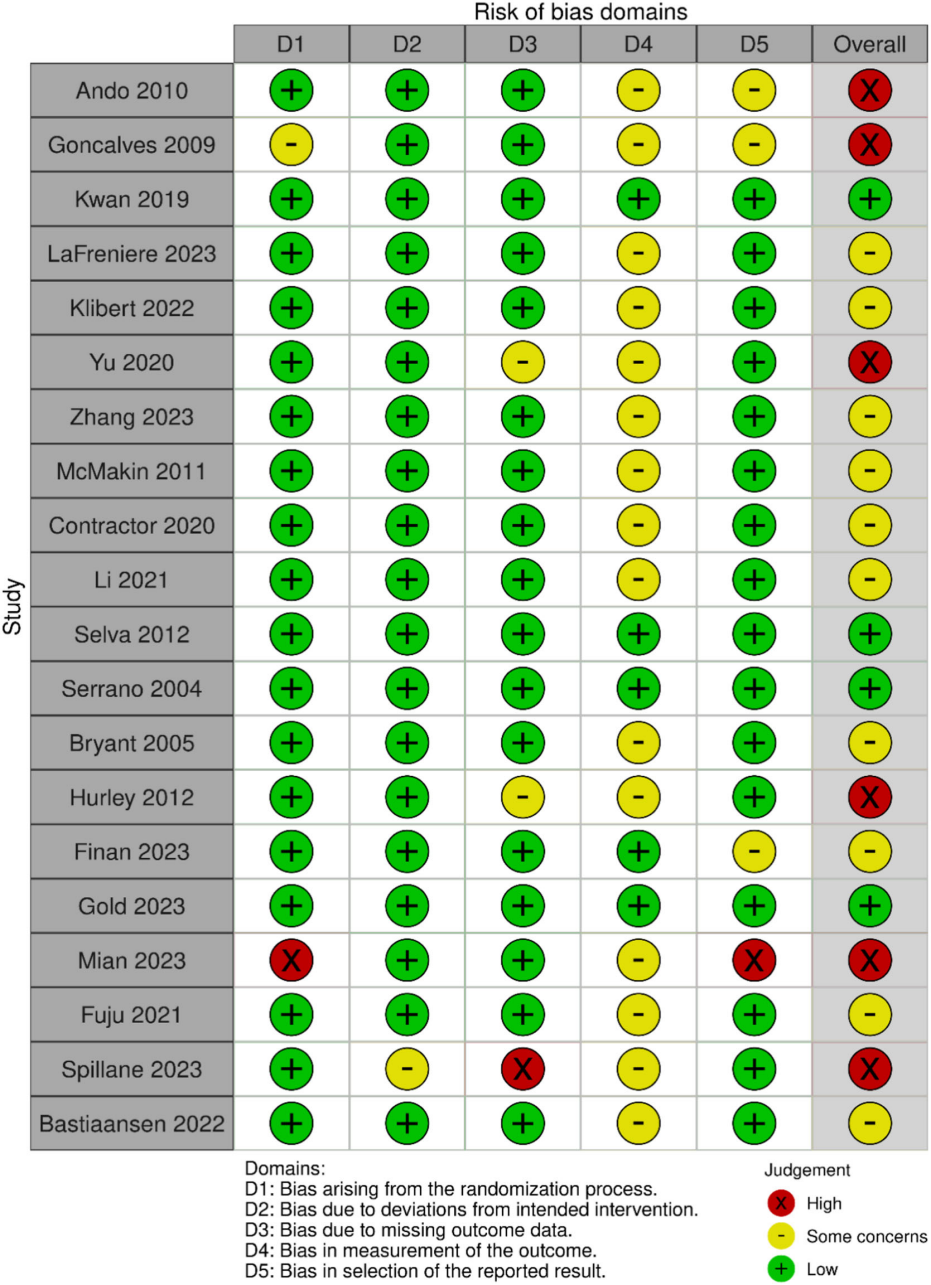
Risk‐of‐bias (RoB 2) traffic‐light plot for included randomized trials (robvis). *Note*: Each row represents an individual study, and each column corresponds to one of the five domains evaluated using the Cochrane Risk of Bias 2 (RoB 2) tool: (D1) bias arising from the randomization process, (D2) bias due to deviations from intended interventions, (D3) bias due to missing outcome data, (D4) bias in measurement of the outcome, and (D5) bias in selection of the reported result. The colors indicate the level of risk: green (“+”) = low risk, yellow (“−”) = some concerns, and red (“×”) = high risk. The final column displays the overall risk‐of‐bias judgment for each study.

Risk of bias was evaluated using the Cochrane Risk of Bias 2 (RoB 2) tool. Four studies (20%) were rated as low risk, 10 studies (50%) as having some concerns, and six studies (30%) as high risk. The domain most frequently rated as “some concerns” was measurement of the outcome, primarily due to reliance on self‐reported measures. However, for studies explicitly stating that outcome assessors were blinded to group allocation or that outcome verification was independently conducted, the risk was rated as low. Figure 2 presents the RoB 2 traffic‐light plot for all included trials, generated with the robvis web application (McGuinness & Higgins, 2021).

The inclusion criteria of this study did not restrict participant age (including adults, adolescents, or children); however, all included samples were from adult populations, with the youngest participants being university students.

### Main meta‐analytic results

**FIGURE 3:**
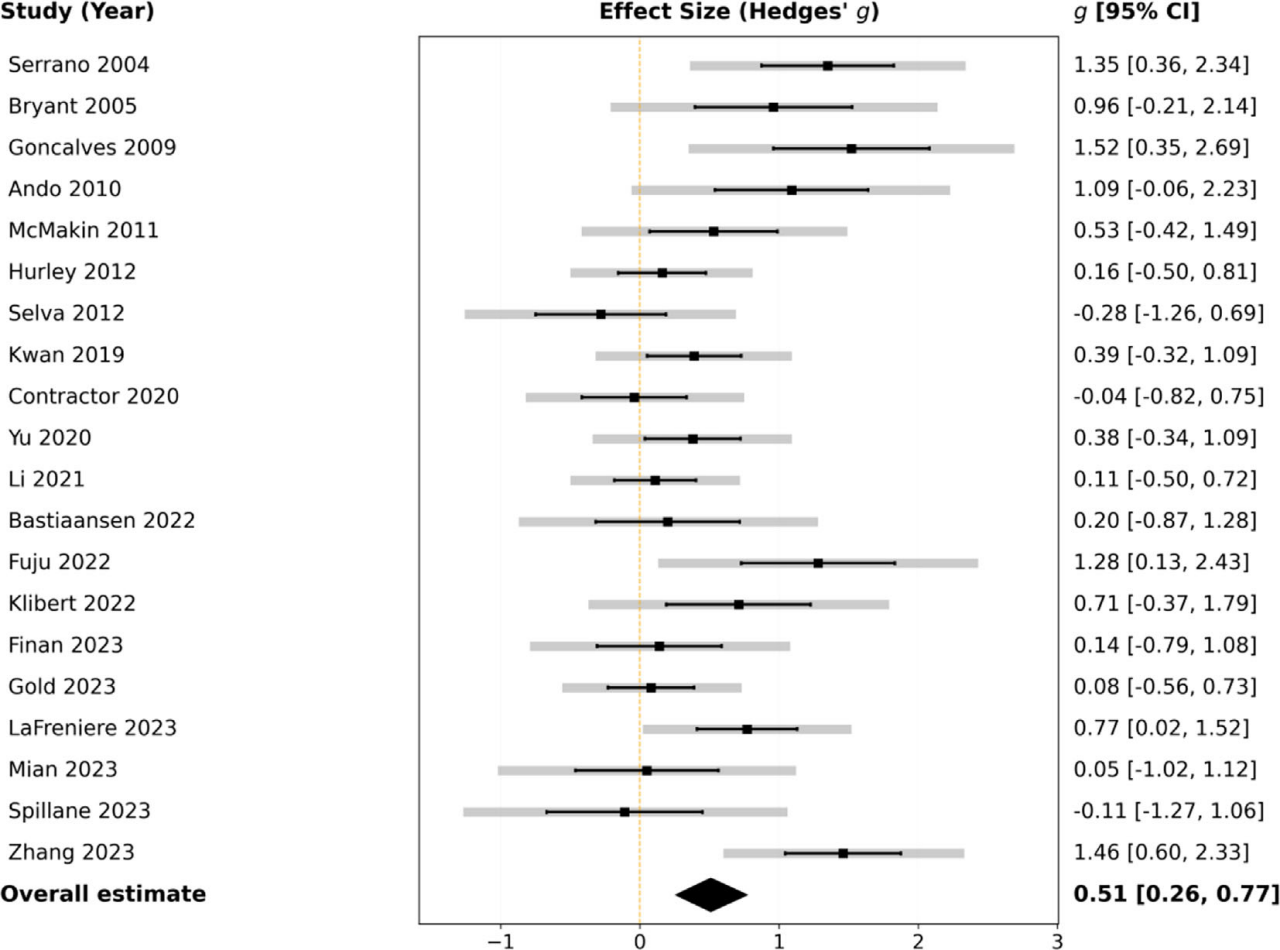
Forest plot of study‐level effect sizes (Hedges' *g*) for savoring interventions. *Note*: Studies are ordered chronologically (2004–2023). Each line represents an individual randomized controlled trial (*K* = 20), displaying the standardized mean difference (Hedges' *g*) and 95% confidence interval for the intervention's effect on emotional outcomes. Black error bars denote sampling precision, whereas gray bars indicate total precision incorporating within‐study variance. The diamond at the bottom shows the overall pooled effect estimated using a three‐level meta‐analysis with cluster‐robust variance estimation (TLMA, CR2), indicating a significant medium‐sized effect (*g* = 0.51, 95% CI [0.26, 0.77], *p* < .001).

The overall pooled effect across all outcomes, presented in Table 2, demonstrated a medium and significant intervention efficacy (*g* = 0.51, 95% CI [0.26, 0.77], *p* < .001) (Figure 3). A decomposition of the variance, detailed in Table 3, revealed substantial heterogeneity (*I* ^2^ = 86.61%). This heterogeneity was predominantly attributable to significant between‐study differences (Level 3: *I* ^2^ = 72.77%, *p* < .001) rather than the significant but smaller within‐study variance across outcomes (Level 2: *I* ^2^ = 13.84%, *p* = .041). The statistical significance of both components, as indicated by LRTs, justifies the use of the three‐level model and provides a strong rationale for the planned moderator analyses.

**TABLE 2:** Overall pooled effect and outcome category moderator analysis.

Analysis	*K*	*k*	*N*	*g*	95% CI	*t*	*df*	*p*
Overall effect	20	45	4805	0.51	[0.26, 0.77]	4.25	18.70	<.001
Outcome category moderator analysis								
Negative emotional disorder	14	17	2875	0.61	[0.31, 0.91]	4.35	15.50	<.001
Negative emotional states	8	8	1214	0.33	[−0.01, 0.68]	2.19	9.30	.056
Positive psychological states	18	20	2311	0.50	[0.24, 0.75]	4.09	17.70	<.001

*Note*: Omnibus test of moderators (CR2‐adjusted): *F*(2, 7.55) = 1.72, *p* = .242. *K* = number of studies; *k* = number of effect sizes; *N* = total participants. All estimates are derived from three‐level random‐effects models. Estimates for the overall effect and moderator analysis are based on models with cluster‐robust variance estimation (CR2) and Satterthwaite degrees of freedom.

**TABLE 3:** Decomposition of variance for the overall effect.

Source of variance	Variance component (*σ* ^2^)	Percentage of total variance (*I* ^2^)	*p* ^a^
Level 3: Between‐studies	0.23	72.77%	<.001
Level 2: Within‐studies	0.04	13.84%	.041
Total heterogeneity	0.27	86.61%	*<.001* ^b^

^a^ Significance was tested using the likelihood ratio test (LRT).

^b^ Total heterogeneity was tested using Cochran's *Q*‐test.

As shown in Table 2, a three‐level meta‐regression with outcome category as a moderator did not detect statistically significant heterogeneity between the subgroups (Wald test *F*(2, 7.55) = 1.72, *p* = .242). For descriptive purposes, the category‐specific pooled estimates were *g* = 0.61 (95% CI [0.31, 0.91], *p* < .001) for negative emotional symptoms, *g* = 0.33 (95% CI [−0.01, 0.68], *p* = .056) for negative emotional states, and *g* = 0.50 (95% CI [0.24, 0.75], *p* < .001) for positive psychological states. Because the omnibus test was nonsignificant, these estimates should not be interpreted as statistically different from one another.

### Moderator analysis

Results of the CR2‐adjusted moderator analyses are presented in Table 4. None of the examined moderators reached statistical significance: risk of bias (Δ*g* = −0.05, *p* = .876), control group type (Δ*g* = −0.47, p = .068), cultural context (Δ*g* = −0.31, *p* = .279), intervention delivery format (*F*(2, 7.03) = 2.96, *p* = .117), and intervention duration (*β* = 0.00, *p* = .818). For descriptive purposes, point estimates were larger for passive controls (*g* = 0.77, 95% CI [0.28, 1.26]) than for active controls (*g* = 0.30, 95% CI [0.04, 0.56]), but this difference did not reach statistical significance. Similarly, savoring interventions showed beneficial point estimates in both Eastern and Western samples, without evidence of a statistically significant difference between cultural contexts.

**TABLE 4:** Moderator analysis summary with CR2 corrections.

Moderator/category	*K*	*k*	*N*	*g*	95% CI	*p*
Risk of bias						
Low/some concerns	14	33	3516	0.53	[0.21, 0.84]	.003
High risk	6	12	1289	0.48	[−0.13, 1.10]	.098
Between‐group test					Δ*g* = −0.05	.876
Control group type						
Passive control	11	24	2699	0.77	[0.28, 1.26]	.007
Active control	9	21	2106	0.30	[0.04, 0.56]	.030
Between‐group test					Δ*g* = −0.47	.068
Cultural context						
Eastern cultures	6	18	1591	0.73	[0.12, 1.34]	.028
Western cultures	14	27	3214	0.42	[0.11, 0.72]	.011
Between‐group test					Δ*g* = −0.31	.279
Intervention delivery format						
Individual	11	23	1061	0.65	[0.25, 1.04]	.005
Online computer‐based	4	12	1953	0.18	[−0.06, 0.41]	.095
Smartphone application	5	10	1791	0.52	[−0.28, 1.32]	.146
Omnibus test					*F*(2, 7.03) = 2.96	.117
Intervention duration (continuous)						
Slope (per day)	20	45	4805	0.00	[−0.03, 0.03]	.818

*Note*: *K* = number of independent studies; *k* = number of effect sizes; *N* = total sample size; *g* = Hedges' *g*; CI = confidence interval; *p* = *p*‐value. Between‐group tests employed cluster‐robust variance estimation (CR2) with small‐sample correction. Δ*g* = difference in effect sizes between subgroups. Omnibus *F*‐tests examine overall moderator significance for categorical variables with >2 levels.

The intervention delivery format did not significantly moderate efficacy, as indicated by the nonsignificant omnibus test (*F*(2, 7.03) = 2.96, *p* = .117). Effect sizes were significant for individual delivery (*g* = 0.65, 95% CI [0.25, 1.04], *p* = .005), with nonsignificant effects for online computer‐based formats (*g* = 0.18, 95% CI [−0.06, 0.41], *p* = .095) and smartphone applications (*g* = 0.52, 95% CI [−0.28, 1.32], *p* = .146). The wide confidence intervals for digital delivery modalities reflected limited statistical power, given the small number of contributing studies. Finally, intervention duration was not significantly associated with effect magnitude (slope *β* = 0.00 per day, 95% CI [−0.03, 0.03], *p* = .818).

### Publication bias assessment

The contour‐enhanced funnel plot was constructed with significance contours centered at an effect size of zero, consistent with the null hypothesis, to facilitate identification of potential questionable research practices.

**FIGURE 4:**
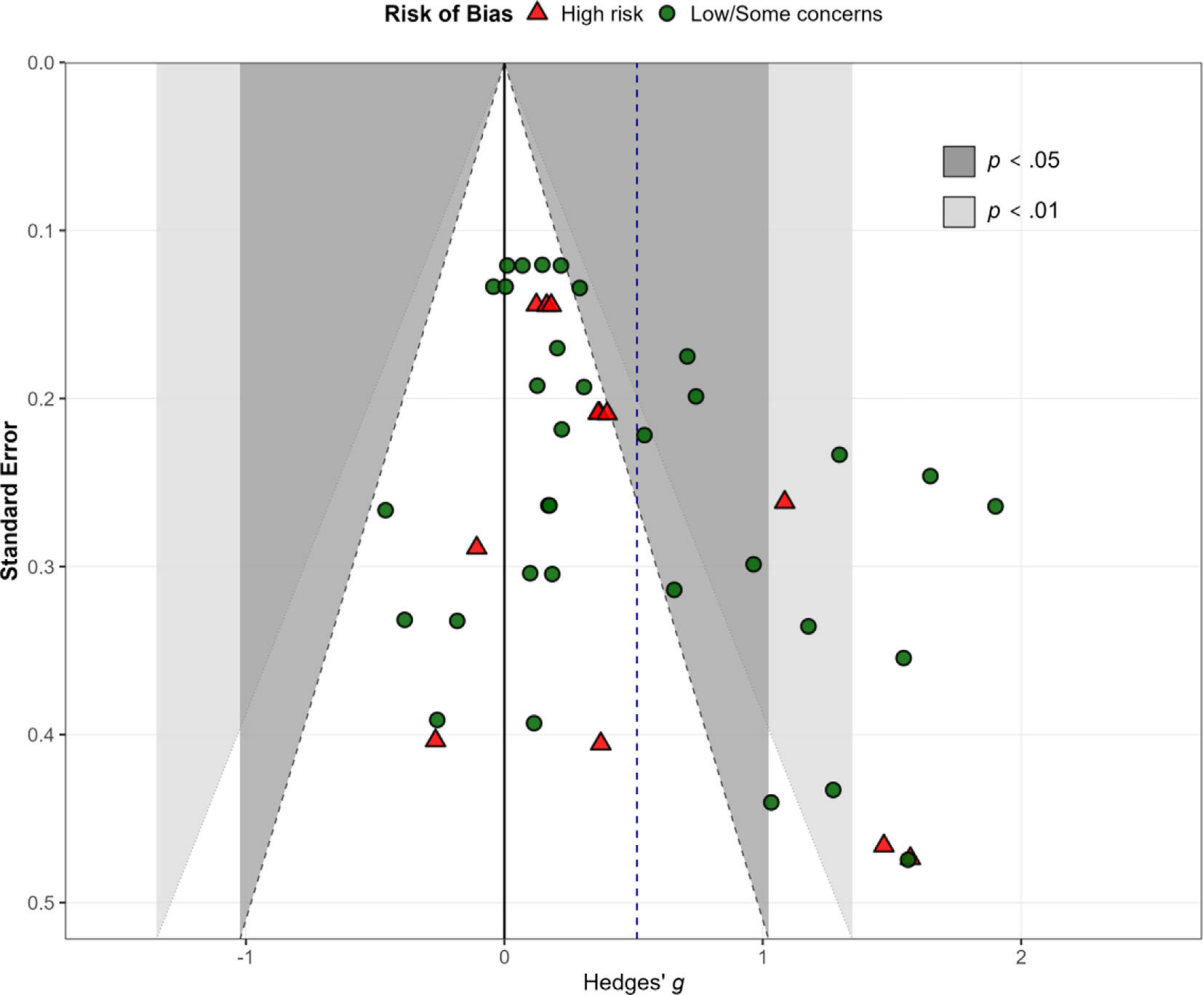
Contour‐enhanced funnel plot for publication bias assessment. *Note*: Each point represents one effect size, plotted by Hedges' *g* (x‐axis) against standard error (y‐axis). The solid black vertical line marks the null effect (*H*_0_: effect = 0), and the dashed blue vertical line indicates the pooled effect estimate (*g* = 0.51). Shaded regions denote two‐sided significance contours (dark gray: *p* < .05; light gray: *p* < .01). The distribution appears relatively symmetric. Egger's regression test (slope for standard error; CR2): *β* = 1.89, *t*(4.84) = 1.97, *p* = .108, suggesting no statistically significant evidence of small‐study effects/publication bias.

Visual inspection of the contour‐enhanced funnel plot revealed no clear asymmetry (Figure 4). Egger's regression test using robust variance estimation (RVE) showed no evidence of small‐study effects, as the slope coefficient for standard error was nonsignificant (*β* = 1.89, *p* = .108). LRTs in both standard and simplified selection models also detected no significant selective reporting bias (LRT = 0.63, *p* = .889; LRT = 0.37, *p* = .542, respectively).

Although PET‐PEESE yielded a near‐zero adjusted estimate (*β* = 0.02, 95% CI [−0.54, 0.57], *p* = .938), this result was interpreted cautiously given the known tendency of PET‐based corrections to over‐adjust toward the null in highly heterogeneous datasets (Stanley, 2017). Instead, bias‐adjusted estimates from trim‐and‐fill (*g* = 0.48, 95% CI [0.24, 0.71]) and selection models (*g* = 0.60 across both specifications) were considered more reliable indicators (Table 5). Taken together, the converging evidence suggests that the observed effect is unlikely to be driven by publication bias and remains robust after adjustment.

**TABLE 5:** Publication bias assessment across multiple correction methods.

Method	Target parameter	Estimate	95% CI	*p*
Egger's test (RVE)	Bias test (slope)	1.89	[NA]	.108
PET‐PEESE (RVE)	Bias‐adjusted effect estimate	0.02	[−0.54, 0.57]	.938
Trim‐and‐fill	Bias‐adjusted effect estimate	0.48	[0.24, 0.71]	<.001
Selection model (standard)	Bias test (LRT)	0.63	[NA]	.889
	Bias‐adjusted effect estimate	0.60	[0.15, 1.06]	.010
Selection model (simplified)	Bias test (LRT)	0.37	[NA]	.542
	Bias‐adjusted effect estimate	0.60	[0.23, 0.97]	.002

*Note*: “Bias Test (Slope)” refers to the coefficient for the standard error in the RVE‐adjusted Egger's regression. “Bias Test (LRT)” refers to the Likelihood Ratio Test statistic comparing the fit of the selection model against the standard random‐effects model. “Bias‐Adjusted Effect Estimate” refers to the pooled effect size estimate after accounting for potential publication bias using the respective method. The PET‐adjusted estimate (derived following the Stanley & Doucouliagos, 2014, decision rule) is presented but should be interpreted with caution, given the test's low statistical power in this sample (k = 20 studies) and the potential for overcorrection towards the null, especially under high heterogeneity (*I* ^*2*^ > 80%). Therefore, the trim‐and‐fill and selection‐model adjusted estimates were considered the primary indicators of the effect magnitude after sensitivity analyses for publication bias.

## DISCUSSION

This meta‐analysis clarified the overall effectiveness of savoring interventions in promoting emotional well‐being. By integrating evidence from RCTs, the findings revealed that savoring interventions significantly enhanced positive emotions and alleviated negative emotional symptoms. Variance decomposition revealed considerable between‐study heterogeneity, warranting further moderator analyses. The moderator analysis indicated that risk of bias, cultural background, intervention format, and intervention duration did not significantly influence intervention effectiveness. Control group type did not reach statistical significance as a moderator (Δ*g* = −0.47, *p* = .068), although point estimates were larger when interventions were compared with passive controls than with active controls. Overall, the interventions demonstrated a moderate pooled effect, suggesting meaningful benefits across diverse research conditions.

Over the past two decades, numerous studies have shown that savoring interventions can enhance positive emotions and reduce depressive or anxiety symptoms (Contractor et al., 2020; LaFreniere & Newman, 2023a; McMakin et al., 2011; Yu et al., 2020) and significantly enhance well‐being (Bryant et al., 2005; Gold et al., 2023; Goncalves et al., 2009; Kwan et al., 2019; Li et al., 2021; Quoidbach et al., 2009).

However, given the diversity of intervention formats and outcome measures, previous research has faced methodological challenges when attempting to integrate quantitative results.

Among the two prior meta‐analyses on savoring interventions, one was limited to student populations, thereby restricting the generalizability of its findings (Zheng et al., 2025); the other deemed quantitative synthesis unfeasible because of the substantial heterogeneity among the included studies (Cullen et al., 2024). To overcome these limitations, the present study adopted a three‐level meta‐analytic approach (TLMA), which accounts for the dependency among multiple effect sizes within a single study. This method decomposes variance into sampling, within‐study, and between‐study levels, enabling a more precise estimation of overall effects, reducing Type I error inflation, and increasing the robustness of moderator analyses (Assink & Wibbelink, 2016).

The results demonstrated that savoring interventions have significant effects on improving emotional well‐being. The pooled effect size (*g* = 0.51) indicates that savoring interventions not only effectively enhance positive emotions but also alleviate negative emotional distress, making them a powerful strategy for promoting mental health. This finding aligns with extensive prior evidence and echoes Bryant's (2021) recent interpretation of savoring theory—that savoring represents a positive emotion regulation process that helps individuals mitigate emotional disorders (e.g. depression and anxiety) while simultaneously promoting positive psychological outcomes such as optimism, happiness, and life satisfaction.

Moreover, in contrast to traditional mindfulness‐based interventions, which typically require an 8‐week structured program with weekly sessions lasting 2–2.5 h led by professionally trained instructors (Keng et al., 2011), savoring interventions are comparatively simpler, more intuitive, and more accessible, demonstrating greater practicality and feasibility. Savoring strategies can be flexibly applied across various everyday contexts (Wallimann et al., 2024), such as walking (Sato et al., 2018), eating (Chee, 2024; Ho et al., 2024), listening to music (Irfan et al., 2022), viewing or taking meaningful photos (Wilson & MacNamara, 2024; Zhang et al., 2023), participating in cultural festivals (Rossetti & Quinn, 2021), or engaging in social activities (Growney et al., 2025). These activities naturally elicit awareness of the present moment and strengthen individuals' ability to regulate positive emotions. Moreover, savoring can be learned through cross‐cultural socialization processes, making it a culturally adaptive positive psychology practice that enhances well‐being (Bryant, 2021).

Looking forward, the integration of virtual reality (VR) technology into savoring practices could offer immersive emotional experiences that strengthen emotion regulation and psychological resilience. Such interdisciplinary applications may assist emerging adults in cultivating mindfulness and positive reflection amid stress and uncertainty and could benefit patients with chronic illnesses such as chronic obstructive pulmonary disease (COPD) by combining relaxation training and savoring exercises to reduce anxiety and improve respiratory functioning (Pancini et al., 2022, 2023).

Furthermore, moderator analyses indicated that the effectiveness of savoring interventions remained stable across methodological variations. Although risk of bias did not significantly moderate outcomes, studies with lower or moderate bias tended to report larger effect sizes: a finding consistent with prior meta‐analyses showing that higher‐quality trials produce smaller, more accurate estimates, whereas lower‐quality studies may overestimate effects due to limitations in randomization, allocation concealment, and blinding (Bolier et al., 2013; Hendriks et al., 2018, 2019). The type of control group also approached significance, with active controls attenuating observed effects (Carr et al., 2020; Hendriks et al., 2018).

Regarding the moderating effect of culture, although both Eastern and Western samples showed significant effect sizes, cultural background did not significantly moderate the intervention outcomes. This finding is consistent with the meta‐analysis by Zheng et al. (2025), indicating that savoring interventions demonstrate relatively stable effects across different cultural contexts. However, the present results differ from those reported by Sieder et al. (2024), who found that mindfulness interventions conducted in Eastern countries were significantly more effective than those in Western countries. The authors suggested that this difference might be related to insufficient sample sizes and lower study quality. Furthermore, Eastern cultures emphasize emotional balance and moderation (Miyamoto & Ma, 2011) and are generally characterized by collectivism, where individuals are more likely to be influenced by group interactions during interventions, potentially giving rise to the Hawthorne effect. In addition, since positive psychology interventions originated in Western contexts, they may be perceived as relatively novel in non‐Western societies, thereby triggering expectancy effects among participants. Combined with the generally lower methodological quality of some non‐Western studies (Hendriks et al., 2018), these factors may collectively contribute to an overestimation of intervention effects.

Finally, no significant differences were found between delivery formats (e.g. individual, online, and mobile‐based), implying strong potential for digital adaptations of savoring interventions. However, this contrasts with findings by Koydemir et al. (2020), who reported smaller effects for technology‐assisted interventions, possibly due to the reduced interpersonal engagement that supports happiness training. Similarly, the duration of interventions was not a significant moderator, though prior meta‐analyses have found that longer programs, typically lasting at least 4 weeks, may yield stronger effects on depression (Bolier et al., 2013) and trends toward improved well‐being (Sin & Lyubomirsky, 2009). These findings suggest that intervention length may influence outcomes differently depending on content, intensity, and participant characteristics.

## LIMITATIONS

Although this study provides important empirical evidence regarding the overall effectiveness and potential moderators of savoring interventions, several limitations should be noted.

First, despite the use of a TLMA to address the dependency among multiple effect sizes within studies and the application of multiple methods (e.g. RVE, trim‐and‐fill, and selection models) to assess publication bias, the potential influence of unpublished or grey literature cannot be fully excluded. Second, the quality of the included studies varied considerably, with approximately 30% classified as having a high risk of bias, which may have led to overestimation of some effect sizes. In addition, most studies relied on self‐report questionnaires to assess positive and negative emotions, making them susceptible to social desirability and recall biases and limiting their ability to reflect objective emotional changes. This measurement approach may also result in common‐method variance (Campbell & Fiske, 1959) and systematic bias, thereby reducing the validity of the findings. Third, although variables such as cultural background, intervention format, and intervention duration did not show significant moderating effects, this may be attributed to the limited sample size and heterogeneity in study designs. In particular, studies involving digital interventions and samples from Eastern cultures remain relatively scarce, and the lack of statistical power may have contributed to the nonsignificant moderation effects. Fourth, this review included only adult participants, restricting the generalizability of findings to adolescents or younger populations. Besides, most interventions were relatively brief (median duration = 14 days), which constrains conclusions regarding long‐term effects. Fifth, this meta‐analysis has not yet examined the moderating effects between healthy and clinical populations. Future research could further compare differences between clinical and nonclinical samples to clarify the applicability of savoring interventions across different health conditions.

Future research should include more cross‐cultural and longitudinal RCTs, particularly those comparing digital and face‐to‐face formats. Integrating physiological or behavioral indicators alongside self‐reports would allow for a more comprehensive, multilevel understanding of the mechanisms and sustained effects of savoring interventions on emotional well‐being.

## CONCLUSION

This study represents the first systematic review and meta‐analysis employing a TLMA to examine the effectiveness of savoring interventions. The results revealed a moderate and significant overall effect (*g* = 0.51, *p* < .001), indicating that savoring interventions can enhance positive psychological states and alleviate negative emotional symptoms. The effectiveness of the interventions was not significantly moderated by cultural background, delivery format, or intervention duration, suggesting cross‐context applicability and practical flexibility. Overall, savoring interventions demonstrated stable psychological benefits under various research conditions.

Compared with other positive psychology interventions, savoring strategies are more closely embedded in daily life, easy to implement and maintain, and effective in enhancing well‐being and reducing symptoms of negative emotional disorders across diverse cultural contexts, demonstrating both theoretical and practical strengths.

In conclusion, savoring interventions represent a cost‐effective, culturally inclusive, and scalable approach to promoting psychological health. Future research could further integrate VR and mobile technologies to develop immersive savoring experiences that enhance emotional regulation and foster innovative practices in mental health promotion.

## CONFLICT OF INTEREST STATEMENT

The authors declare no conflicts of interest.

## ETHICS STATEMENT

The review protocol was prospectively registered with INPLASY (registration number: INPLASY202530114).

## SUPPORTING INFORMATION

The Supporting Information includes the GROOVE overlap analysis, PRISMA checklist, full search strategies, reasons for study exclusion, and detailed study characteristics (Tables S1–S5).

## Supporting information


**Table S1** Summary comparison of previous meta‐analyses and the present study.
Table S2 PRISMA checklist.
Table S3 Keywords and search results in different databases.
Table S4 Excluded studies and reasons.
Table S5 Details of data extraction from included randomized controlled trials.

## Data Availability

The original contributions are provided in the article and Supporting Information. Further inquiries can be directed to the corresponding author.
