# Supplementary material for: Effectiveness of savoring interventions: A systematic review and meta‐analysis of randomized controlled trials
Source: Appl Psychol Health Well Being. 2026 Mar 8;18(2):e70134. doi: 10.1111/aphw.70134 (PMC12968602; doi:10.1111/aphw.70134)
Supplement: Supplementary file 1 — Table S1 Summary comparison of previous meta‐analyses and the present study. Table S2 PRISMA checklist. Table S3 Keywords and search results in different databases. Table S4 Excluded studies and reasons. Table S5 Details of data extraction from included randomized controlled trials. [file APHW-18-0-s001.docx]

**Table S1**

*Summary Comparison of Previous Meta-Analyses and the Present Study*

|  | Previous Meta-Analyses | Current Meta-Analysis |
| --- | --- | --- |
| Title | Examining the Influence of Savoring Interventions on Positive Emotions in University Students: A Systematic Review and Meta-Analysis | Effectiveness of Savoring Interventions: A Systematic Review  and Meta-analysis of Randomized Controlled Trials |
| Literature search periods | From 1998 ( the start of the positive psychology movement ) to February 16, 2023 | From inception till March 6, 2025 |
| Search databases | Web of Science, Scopus, PubMed, and PsycINFO | PubMed, PsycINFO, Cochrane Library, CINAHL, MEDLINE, and Google scholar |
| Numbers of included studies | 14 studies | 20 studies |
| PICOs | P: university or college students  I: structured activities designed to enhance individuals' ability to savor positive experiences  C: received no intervention or alternative interventions  O: emotional well-being  S: randomized controlled trials | P: human participants  I: interventions or approaches that enhanced a person’s capacity to appreciate and regulate positive experiences via a deliberate  focus on positive emotional feedings and conscious effort to identify, amplify, prolong, or meaningfully engage with pleasurable moments across past, present, and future timeframes  C: received no intervention or alternative interventions  O: positive and negative emotional outcomes  S: randomized controlled trials |
| Result: summary effect sizes (Hedges’g with 95% CIs) | positive emotions: *g* = 0.706 (95% CI: 0.477 ~ 0.935), *p* <.001 | 1. overall effect: *g* = 0.51 (95% CI: 0.26~0.77), *p* <.001  2. negative emotional disorder: *g* = 0.61 (95% CI: 0.31~0.91), *p* <.001  3. negative emotional states: *g* = -0.33 (95% CI: -0.01~-0.68), *p* =.056  4. positive psychological states: *g* = -0.50 (95% CI: 0.24~0.75), *p* <.001 |
| Estimation of effect size heterogeneity (*I^2^*) | I ^2^= 59.922, *p* <.01 | 1. Level 3: between-studies: I^2^ = 72.77%, *p* <.001  2. Level 2: within-studies: I^2^ = 13.84%, *p* =.041  3. Total heterogeneity: I^2^ = 86.61%, *p* <.001 |
| Identified moderators | 1.Culture (Western/Nonwestern): Q = .425, *p* = 0.514  2.Intervention type (past/present/future): Q = 0.359, *p* = .836 | 1. Risk of Bias: Δg = -0.05, p = .876  2. Control Group Type: Δg = -0.47, *p* = .068  3. Culture Context: Δg = -0.31, p = .279  4. Intervention delivery Format: F(2, 7.03) = 2.96, *p* = .117  5. Intervention Duration: β = .00, *p* = .818 |

*Note. Δg = difference in effect sizes between subgroups*

**Table S2**

*PRISMA Checklist*

| **TITLE** | | |  |
| --- | --- | --- | --- |
| Title | 1 | Identify the report as a systematic review. | Title |
| **ABSTRACT** | | |  |
| Abstract | 2 | See the PRISMA 2020 for Abstracts checklist. | Abstract |
| **INTRODUCTION** | | |  |
| Rationale | 3 | Describe the rationale for the review in the context of existing knowledge. | Introduction |
| Objectives | 4 | Provide an explicit statement of the objective(s) or question(s) the review addresses. | Introduction |
| **METHODS** | | |  |
| Eligibility criteria | 5 | Specify the inclusion and exclusion criteria for the review and how studies were grouped for the syntheses. | Methods |
| Information sources | 6 | Specify all databases, registers, websites, organisations, reference lists and other sources searched or consulted to identify studies. Specify the date when each source was last searched or consulted. | Methods |
| Search strategy | 7 | Present the full search strategies for all databases, registers and websites, including any filters and limits used. | Methods, Table S3 |
| Selection process | 8 | Specify the methods used to decide whether a study met the inclusion criteria of the review, including how many reviewers screened each record and each report retrieved, whether they worked independently, and if applicable, details of automation tools used in the process. | Methods |
| Data collection process | 9 | Specify the methods used to collect data from reports, including how many reviewers collected data from each report, whether they worked independently, any processes for obtaining or confirming data from study investigators, and if applicable, details of automation tools used in the process. | Methods |
| Data items | 10a | List and define all outcomes for which data were sought. Specify whether all results that were compatible with each outcome domain in each study were sought (e.g., for all measures, time points, analyses), and if not, the methods used to decide which results to collect. | Methods  Table 1 |
|  | 10b | List and define all other variables for which data were sought (e.g., participant and intervention characteristics, funding sources). Describe any assumptions made about any missing or unclear information. | Methods |
| Study risk of bias assessment | 11 | Specify the methods used to assess risk of bias in the included studies, including details of the tool(s) used, how many reviewers assessed each study and whether they worked independently, and if applicable, details of automation tools used in the process. | Methods |
| Effect measures | 12 | Specify for each outcome the effect measure(s) (e.g., risk ratio, mean difference) used in the synthesis or presentation of results. | Methods |
| Synthesis methods | 13a | Describe the processes used to decide which studies were eligible for each synthesis (e.g., tabulating the study intervention characteristics and comparing against the planned groups for each synthesis (item #5)). | Methods, Table 1 |
|  | 13b | Describe any methods required to prepare the data for presentation or synthesis, such as handling of missing summary statistics, or data conversions. | Methods |
|  | 13c | Describe any methods used to tabulate or visually display results of individual studies and syntheses. | Methods, Table 2, 3 |
|  | 13d | Describe any methods used to synthesize results and provide a rationale for the choice(s). If meta-analysis was performed, describe the model(s), method(s) to identify the presence and extent of statistical heterogeneity, and software package(s) used. | Methods |
|  | 13e | Describe any methods used to explore possible causes of heterogeneity among study results (e.g., subgroup analysis, meta-regression). | Methods |
|  | 13f | Describe any sensitivity analyses conducted to assess robustness of the synthesized results. | Methods |
| Reporting bias assessment | 14 | Describe any methods used to assess risk of bias due to missing results in a synthesis (arising from reporting biases). | Methods |
| Certainty assessment | 15 | Describe any methods used to assess certainty (or confidence) in the body of evidence for an outcome. | Methods |
| **RESULTS** | | |  |
| Study selection | 16a | Describe the results of the search and selection process, from the number of records identified in the search to the number of studies included in the review, ideally using a flow diagram. | Results, Figure 2 |
|  | 16b | Cite studies that might appear to meet the inclusion criteria, but which were excluded, and explain why they were excluded. | Results, Table S4 |
| Study characteristics | 17 | Cite each included study and present its characteristics. | Results, Table 1 |
| Risk of bias | 18 | Present assessments of risk of bias for each included study. | Figure 3, Table 1 |
| Results of individual studies | 19 | For all outcomes, present, for each study: (a) summary statistics for each group (where appropriate) and (b) an effect estimates and its precision (e.g., confidence/credible interval), ideally using structured tables or plots. | Results, Figure 4,  Table 2, Table 3 |
| Results of syntheses | 20a | For each synthesis, briefly summarise the characteristics and risk of bias among contributing studies. | Results, Table 1 |
|  | 20b | Present results of all statistical syntheses conducted. If meta-analysis was done, present for each the summary estimate and its precision (e.g., confidence/credible interval) and measures of statistical heterogeneity. If comparing groups, describe the direction of the effect. | Results, Figure 4, Table 2 ~ Table 4 |
|  | 20c | Present results of all investigations of possible causes of heterogeneity among study results. | Results, Table 3 |
|  | 20d | Present results of all sensitivity analyses conducted to assess the robustness of the synthesized results. | Results |
| Reporting biases | 21 | Present assessments of risk of bias due to missing results (arising from reporting biases) for each synthesis assessed. | Results, Figure 3 Table 1 |
| Certainty of evidence | 22 | Present assessments of certainty (or confidence) in the body of evidence for each outcome assessed. | Results, Figure 4, Table 2 ~ Table 4 |
| **DISCUSSION** | | |  |
| Discussion | 23a | Provide a general interpretation of the results in the context of other evidence. | Discussion |
|  | 23b | Discuss any limitations of the evidence included in the review. | Discussion |
|  | 23c | Discuss any limitations of the review processes used. | Discussion |
|  | 23d | Discuss implications of the results for practice, policy, and future research. | Discussion |
| **OTHER INFORMATION** | | |  |
| Registration and protocol | 24a | Provide registration information for the review, including register name and registration number, or state that the review was not registered. | Methods |
|  | 24b | Indicate where the review protocol can be accessed, or state that a protocol was not prepared. | Methods, |
|  | 24c | Describe and explain any amendments to information provided at registration or in the protocol. | Methods |
| Support | 25 | Describe sources of financial or non-financial support for the review, and the role of the funders or sponsors in the review. | Funding |
| Competing interests | 26 | Declare any competing interests of review authors. | Conflicts of Interest |
| Availability of data, code and other materials | 27 | Report which of the following are publicly available and where they can be found: template data collection forms; data extracted from included studies; data used for all analyses; analytic code; any other materials used in the review. | Results, Table S1S5 |

**Table S3**

*Keywords and Search Results in Different Databases*

| Database | Keyword | Filter | Date | Results |
| --- | --- | --- | --- | --- |
| PubMed | (“savoring the moment” OR “savoring the moment” OR “savoring intervention” OR “savoring intervention” OR “savoring the moment” OR “savoring the moment” OR “three good thing*” OR “self-congratulations” OR “memory building” OR “positive emotion regulation” OR “positive life review”) AND (“randomized controlled trial*” OR “RCT” OR “clinical trial*”) AND (“positive emotion*” OR “positive affect*” OR “well-being” OR “wellbeing” OR “spiritual well-being” OR “happiness” OR “PANAS” OR “depression” OR “anxiety”) | Title Abstract  Keyword  Randomized Controlled Trial | March 6, 2025 | 1076 |
| CINAHL | (“savoring the moment” OR “savoring the moment” OR “savoring intervention” OR “savoring intervention” OR “savoring the moment” OR “savoring the moment” OR “three good thing*” OR “self-congratulations” OR “memory building” OR “positive emotion regulation” OR “positive life review”) AND (“randomized controlled trial*” OR "RCT" OR “clinical trial*”) AND (“positive emotion*” OR “positive affect*” OR “well-being” OR “wellbeing” OR “spiritual well-being” OR “happiness” OR “PANAS” OR “depression” OR “anxiety”) | Title Abstract  Keyword  Randomized Controlled Trial | March 6, 2025 | 15 |
| Cochrane CENTRAL | (“savoring the moment” OR “savoring the moment” OR “savoring intervention” OR “savoring intervention” OR “savoring the moment” OR “savoring the moment” OR “three good thing*” OR “self-congratulations” OR “memory building” OR “positive emotion regulation” OR “positive life review”) AND (“randomized controlled trial*” OR “RCT” OR “clinical trial*”) AND (“positive emotion*” OR “positive affect*” OR “well-being” OR “wellbeing” OR “spiritual well-being” OR “happiness” OR “PANAS” OR “depression” OR “anxiety”) | Title Abstract  Keyword | J March 6, 2025 | 51 |
| psycINFO | (“savoring the moment” OR “savoring the moment” OR “savoring intervention” OR “savoring intervention” OR “savoring the moment” OR “savoring the moment” OR “three good thing*” OR “self-congratulations” OR “memory building” OR “positive emotion regulation” OR “positive life review”) AND (“randomized controlled trial*” OR “RCT” OR “clinical trial*”) AND (“positive emotion*” OR “positive affect*” OR “well-being” OR “wellbeing” OR “spiritual well-being” OR “happiness” OR “PANAS” OR “depression” OR “anxiety”) | Title Abstract  Keyword  Randomized Controlled Trial | March 6, 2025 | 10 |
| MEDLINE | (“savoring the moment” OR “savoring the moment” OR “savoring intervention” OR “savoring intervention” OR “savoring the moment” OR “savoring the moment” OR “three good thing*” OR “self-congratulations" OR "memory building” OR “positive emotion regulation” OR “positive life review”) AND (“randomized controlled trial*” OR “RCT” OR “clinical trial*”) AND (“positive emotion*”OR “positive affect*” OR “well-being” OR “wellbeing” OR “spiritual well-being” OR “happiness” OR “PANAS” OR “depression” OR “anxiety”) | Title Abstract  Keyword  Randomized Controlled Trial | March 6, 2025 | 33 |

NA: not applied

**Table S4**

*Excluded Studies and Reasons*

| Citations | Reasons |
| --- | --- |
| Cline, M., Roberts, P., Werlau, T., Hauser, P., & Smith-Miller, C. (2022). Three good things: Promote work-life balance, reduce burnout, enhance reflection among newly licensed RNs. *Nursing Forum, 57*(6), 1390–1398. https://doi.org/10.1111/nuf.12830 | Not a randomized trial |
| Tighe, C. A., Dautovich, N. D., Hilgeman, M. M., & Allen, R. S. (2022). Links between savoring, rumination, and sleep-related  experiences across Adulthood: Implications for older adults. *Clinical Gerontologist, 45*(2), 419–429.  https://doi.org/10.1080/07317115.2021.1878404 | Not a randomized trial |
| Smith, J. L., & Hollinger-Smith, L. (2015). Savoring, resilience, and psychological well-being in older adults. *Aging & Mental*  *Health, 19*(3), 192–200. https://doi.org/10.1080/13607863.2014.986647 | Not a randomized trial |
| Smith, J. L., & Hanni, A. A. (2019). Effects of a savoring intervention on resilience and well-being of older adults. *Journal of Applied*  *Gerontology: The Official Journal of the Southern Gerontological Society*, *38*(1), 137–152.  https://doi.org/10.1177/0733464817693375 | Not a randomized trial |
| Villani, D., Pancini, E., Pesce, F., & Scuzzarella, L. (2023). Savoring life during pandemic: An online intervention to promote well  being in emerging adults. *BMC Psychology, 11*(1), 196. https://doi.org/10.1186/s40359-023-01225-z | Not a randomized trial |
| Lyubomirsky, S., Tkach, C., & DiMatteo, M. R. (2005). What are the Differences between Happiness and Self-Esteem. *Social*  *Indicators Research*, *78*(3), 363404. https://doi.org/10.1007/s11205-005-0213-y | Non-savoring intervention study |
| Passmore, H.-A., & Howell, A. J. (2014). Eco-existential positive psychology: Experiences in nature, existential anxieties, and well-  being. *The Humanistic Psychologist*, *42*(4),370–388. https://doi.org/10.1080/08873267.2014.920335 | Non-savoring intervention study |
| Ho, H. C., Lai, A. Y., Mui, M. W., Wan, A., Yew, C. W., & Lam, T. H. (2024). A cluster randomized controlled trial of a brief positive  healthy eating intervention. *Journal of Health Psychology*, *29*(11), 1210–1227.  https://doi.org/10.1177/13591053231225934 | Focus on positive healthy eating intervention rather than savoring intervention |
| Pancini, E., Villani, D., & Riva, G. (2022). The e-SaVoR Project: Savoring and virtual reality to enhance emerging adults’ well-being.  *Cyberpsychology, Behavior and Social Networking*, *25*(12), 834–835.  https://doi.org/10.1089/cyber.2022.29261.ceu | Protocol |
| Pancini, E., Villani, D., & Riva, G. (2023). oVeRcomING COPD: Virtual reality and savoring to promote the well-Being of patients  with chronic obstructive pulmonary disease. *Cyberpsychology, Behavior and Social Networking*, *26*(1), 65–67.  https://doi.org/10.1089/cyber.2022.29265.ceu | Protocol |
| LaFreniere, L. S., & Newman, M. G. (2024). Savoring changes novel positive mindset targets of GAD treatment: Optimism,  prioritizing positivity, kill-joy thinking, and worry mediation. *Behavior Research and Therapy, 177*, 104541.  https://doi.org/10.1016/j.brat.2024.104541 | Overlapping participants with the author’s another publication, which is already enrolled in our meta-analysis |
| Seligman, M. E., Steen, T. A., Park, N., & Peterson, C. (2005). Positive psychology progress: Empirical validation of interventions.  *The American Psychologist, 60*(5), 410–421. https://doi.org/10.1037/0003-066X.60.5.410 | The experimental group in this study included five interventions: gratitude visit, you at your best, three good things in life, using signature strengths in a new way, and identifying signature strengths |
| Ho, H. C., Mui, M., Wan, A., Ng, Y. L., Stewart, S. M., Yew, C., Lam, T. H., & Chan, S. S. (2016a). Happy Family Kitchen II: A cluster randomized controlled trial of a community-based family intervention for enhancing family communication and well-being in Hong Kong. *Frontiers in Psychology, 7*, 638. https://doi.org/10.3389/fpsyg.2016.00638 | The experimental group in this study received five positive interventions: joy, gratitude, flow, savoring, and listening |

| Citations | Reasons |
| --- | --- |
| Ho, H. C., Mui, M., Wan, A., Ng, Y. L., Stewart, S. M., Yew, C., Lam, T. H., & Chan, S. S. (2016b). Happy Family Kitchen II: A  cluster randomized controlled trial of a community-based positive psychology family intervention for subjective happiness and  health-related quality of life in Hong Kong. *Trials, 17,* 367. https://doi.org/10.1186/s13063-016-1508-9 | The experimental group in this study received five positive interventions: joy, gratitude, flow, savoring, and listening |
| Moskowitz, J. T., Cheung, E. O., Snowberg, K. E., Verstaen, A., Merrilees, J., Salsman, J. M., & Dowling, G. A. (2019). Randomized  controlled trial of a facilitated online positive emotion regulation intervention for dementia caregivers. *Health*  *Psychology: Official Journal of the Division of Health Psychology, American Psychological Association, 38*(5), 391–402.  https://doi.org/10.1037/hea0000680 | This study combines five interventions: savoring, mindfulness, positive reappraisal, personal strengths and attainable goal setting, and kindness, collectively referred to as LEAF |
| Ong, A. D., Moskowitz, J. T., Wethington, E., Addington, E. L., Sanni, M., Goktas, S., Sluys, E., Swong, S., Kim, P., & Reid, M. C.  (2022). Lessons in Affect Regulation to Keep Stress and Pain UndeR control (LARKSPUR): Design of a randomized controlled  trial to increase positive affect in middle-aged and older adults with fibromyalgia. *Contemporary Clinical Trials, 120*, 106880.  https://doi.org/10.1016/j.cct.2022.106880 | This study combines eight positive interventions: noticing positive events, savoring positive events, identifying personal strengths, behavioral activation to set and work toward attainable foals, mindfulness, positive reappraisal, gratitude, and kindness, collectively referred to as LAPKSPUR |
| Garland, E. L., Hanley, A. W., Nakamura, Y., Barrett, J. W., Baker, A. K., Reese, S. E., Riquino, M. R., Froeliger, B., & Donaldson, G.  W. (2022). Mindfulness-oriented recovery enhancement vs supportive group therapy for co-occurring opioid misuse and chronic  pain in primary care: A randomized clinical trial. *JAMA Internal Medicine, 182*(4), 407–417.  https://doi.org/10.1001/jamainternmed.2022.0033 | This study combines three interventions: mindfulness, reappraisal, and savoring, collectively referred to as MORE |
| Garland, E. L., Nakamura, Y., Bryan, C. J., Hanley, A. W., Parisi, A., Froeliger, B., Marchand, W. R., & Donaldson, G. W. (2024).  Mindfulness-oriented recovery enhancement for veterans and military personnel on long-term opioid Therapy for chronic pain: A randomized clinical trial. *The American Journal of Psychiatry, 181*(2), 125–134. https://doi.org/10.1176/appi.ajp.20230272 | This study combines three interventions: mindfulness, reappraisal, and savoring, collectively referred to as MORE |
| Kumar, D., Corner, S., Kim, R., & Meuret, A. (2024). A randomized controlled trial of brief behavioral activation plus savoring for positive affect dysregulation in university students. *Behavior Research and Therapy, 177*, 104525. https://doi.org/10.1016/j.brat.2024.104525 | The experimental group in this study received two interventions: brief behavioral activation and savoring |
| Quoidbach, J., Wood, A. M., & Hansenne, M. (2009). Back to the future: The effect of daily practice of mental time travel into the future on happiness and anxiety. *The Journal of Positive Psychology, 4*(5), 349–355. https://doi.org/10.1080/17439760902992365 | Lack of pre-test and post-test SHS and STAI scores for each group |
| Frein, S. T., & Ponsler, K. (2013). Increasing positive affect in college students. *Applied Research in Quality of Life, 9*(1), 1–13. https://doi.org/10.1007/s11482-013-9210-5 | The number of participants in each group was not reported |
| Smith, J. L., & Bryant, F. B. (2019). Enhancing positive perceptions of aging by savoring life lessons. *Aging & Mental Health, 23*(6), 762–770. https://doi.org/10.1080/13607863.2018.1450840 | Only estimated marginal means (SEs) were found in each group |
| Borelli, J. L., Kerr, M. L., Smiley, P. A., Rasmussen, H. F., Hecht, H. K., & Campos, B. (2023). Relational savoring intervention: Positive impacts for mothers and evidence of cultural compatibility for Latinas. *Emotion (Washington, D.C.), 23*(2), 303–320. https://doi.org/10.1037/emo0001102 | Only effect size magnitude was found in each group |

| Citations | Reasons |
| --- | --- |
| Rosen, F. N., & LaFreniere, L. S. (2023). Savoring, worry, and positive emotion duration in generalized anxiety disorder: Assessment and interventional experiment. *Journal of Anxiety Disorders, 97*, 102724. https://doi.org/10.1016/j.janxdis.2023.102724 | The number of participants in each group was not reported |
| Zehner, N., Polding, L., Faraci Sindra, V., & Shieh, L. (2023). Prospective pilot study of the Three Good Things positive psychology intervention in short-term stay hospitalised patients. *Postgraduate Medical Journal, 99*(1170), 302–307.  https://doi.org/10.1136/postgradmedj-2021-141010 | The experimental results are presented as medians rather than means or standard deviations |
| Straszewski, T., & Siegel, J. T. (2018). Positive Emotion Infusions: Can Savoring Increase Help-Seeking Intentions among People with Depression? *Appl Psychol Health Well Being*, *10*(1), 171–190. https://doi.org/10.1111/aphw.12122 | Focus on help-seeking intentions rather than emotional outcome measure |
| Borelli, J. L., Bond, D. K., Fox, S., & Horn-Mallers, M. (2020). Relational Savoring Reduces Physiological Reactivity and Enhances Psychological Agency in Older Adults. *J Appl Gerontol*, *39*(3), 332–342. https://doi.org/10.1177/0733464819866972 | Focus on physiological reactivity and psychological agency rather than emotional outcome measure |
| Irvin, K. M., Bell, D. J., Steinley, D., & Bartholow, B. D. (2022). The thrill of victory: Savoring positive affect, psychophysiological reward processing, and symptoms of depression. *Emotion*, *22*(6), 1281–1293.  https://doi.org/10.1037/emo0000914 | Focus on neurophysiological reactivity rather than emotional outcomes measure |
| Borelli, J. L., Kazmierski, K. F. M., Gaskin, G. E., Kerr, M. L., Smiley, P. A., & Rasmussen, H. F. (2023). Savoring interventions for mothers of young children: Mechanisms linking relational savoring and personal savoring to reflective functioning. *Infant Ment Health J*, *44*(2), 200–217. https://doi.org/10.1002/imhj.22038 | Focus on reflective functioning of mother rather than emotional outcome measure |
| Cheng, Y., Peters, B. R., & MacNamara, A. (2023). Positive emotion up-regulation is resistant to working memory load: An electrocortical investigation of reappraisal and savoring. *Psychophysiology*, *60*(12), e14385. https://doi.org/10.1111/psyp.14385 | Focus on working memory load rather than emotional outcome measure |
| Palmer, A. M., Carpenter, M. J., Baker, N. L., Froeliger, B., Foster, M. G., Garland, E. L., Saladin, M. E., & Toll, B. A. (2024). Development of two novel treatments to promote smoking cessation: Savor and retrieval-extinction training pilot clinical trial findings. *Exp Clin Psychopharmacol*, *32*(1), 16–26. https://doi.org/10.1037/pha0000644 | Focus on smoking cessation rather than emotional outcome measure |
| LaFreniere, L. S., & Newman, M. G. (2023). Reducing contrast avoidance in GAD by savoring positive emotions: Outcome and mediation in a randomized controlled trial. *J Anxiety Disord*, *93*, 102659. https://doi.org/10.1016/j.janxdis.2022.102659 | 1.Overlapping participants with the author’s another publication, which is already enrolled in our meta-analysis  2.Focus on contrast avoidance rather than emotional outcome measure |
| Borelli, J. L., Perzolli, S., Kerr, M., & Smiley, P. A. (2024). Predicting fidelity and treatment outcomes in savoringinterventions among mothers of young children. *Infant Ment Health J.* https://doi.org/10.1002/imhj.22130 | Focus on fidelity and treatment outcomes rather than emotional outcome measure |
| Smiley, P. A., Ahn, A., Blackard, M. B., Borelli, J. L., & Doan, S. N. (2024). Undoing mothers' avoidant coping with children's negative emotion: A randomized controlled trial of relational savoring. *J Fam Psychol*, *38*(3), 365–376. https://doi.org/10.1037/fam0001186 | Focus on undoing mother’s avoidant coping rather than emotional outcome measure |
| Ouweneel, E., Le Blanc, P. M., & Schaufeli, W. B. (2014). On being grateful and kind: Results of two randomized controlled trials on study-related emotions and academic engagement. *The Journal of Psychology, 148*(1), 37–60. https://doi.org/10.1080/00223980.2012.742854 | Study focused on gratitude and kindness, |
| Sheldon, K. M., & Lyubomirsky, S. (2006). How to increase and sustain positive emotion: The effects of expressing gratitude and visualizing best possible selves. *The Journal of Positive Psychology, 1*(2), 73–82. https://doi.org/10.1080/17439760500510676 | Study focused on gratitude and best possible self-interventions |
| Deng, Y. H., Yijie, Z., Li, Y., & X., L. (2016). Effects of gratitude-based intervention on aggression and subjective well-being in violent criminals. *Chinese Journal of Clinical Psychology, 24*(2), 368–372. https://doi.org/10.16128/j.cnki.1005-3611.2016.02.042 | Study focused on gratitude |

| Citations | Reasons |
| --- | --- |
| Datu, J. A. D., Valdez, J. P. M., McInerney, D. M., & Cayubit, R. F. (2022). The effects of gratitude and kindness on life satisfaction,  positive emotions, negative emotions, and COVID-19 anxiety: An online pilot experimental study. *Applied Psychology. Health*  *and Well-Being, 14*(2), 347–361. https://doi.org/10.1111/aphw.12306 | Study focused on gratitude and kindness |
| Nicolson, N. A., Peters, M. L., & In den Bosch-Meevissen, Y. M. C. (2020). Imagining a positive future reduces cortisol response to  awakening and reactivity to acute stress. *Psychoneuroendocrinology, 116*, 104677.  https://doi.org/10.1016/j.psyneuen.2020.104677 | Study focused on best possible self-interventions |

**Table S5**

*Details of Data Extraction from Included Randomized Controlled Trials*

| First Author & Year | Details of data extraction from included trials |
| --- | --- |
| Ando, 2010 | 1.Culture was extracted from Patients and Methods; duration, control, delivery format and arms were extracted from Interventions  2.The HADS scores were extracted from Table 2. |
| Gonçalves, 2009 | 1.Culture was extracted from Methods; duration, control, delivery format and arms were extracted from Interventions   1. 2.The GDS and LSI scores were extracted from Results. |
| Kwan, 2019 | 1.Culture was extracted from Methods; duration, control, delivery format and arms were extracted from Interventions  2.The MQOL-HK and HADS scores were extracted from Table 4. |
| LaFreniere, 2023 | 1.Culture was extracted from author’s information; duration, control and arms were extracted from Method   1. 2.The PANAS-X, BDI-II, and PSWQ scores were extracted from Table 1. |
| Kliber, 2022 | 1.Culture was extracted from Participants; duration, control, delivery format and arms were extracted from Interventions  3.The PANAS-PA scores were extracted from Table 1. |
| Yu, 2020 | 1.Culture and duration were extracted from Participants; duration, control, delivery format and arms were extracted from Procedure   1. 2.The PANAS and CES-D scores were extracted from Table 1. |
| Zhang, 2023 | 1.Culture was extracted from author’s information; duration, control, delivery format and arms were extracted from Procedures   1. 2.The PANAS scores were extracted from Table 2. |
| McMakin, 2011 | 1.Culture, duration, control, delivery format and arms were extracted from Procedure   1. 2.The PANAS and BDI-II scores were extracted from Table 1. |
| Contractor, 2020 | 1.Culture, control, duration, delivery format and arms were extracted from Procedure and Participants  2.The PANAS and PHQ-9 scores were extracted from Table 1. |
| Li, 2021 | 1.Culture was extracted from Participants; duration, control, delivery format and arms were extracted from Procedure   1. 2.The depression and anxiety scale scores were extracted from Table 2. 2. 3.The PANAS, CES-D, SHS, and GAD scores were extracted from Table 3. |
| Selva, 2012 | 1.Culture was extracted from Participants and design; duration, control, delivery format and arms were extracted from Procedure   1. 2.The GDS and LSIA scores were extracted from Table 1. |
| Serrano, 2004 | 1.Culture was extracted from Participants; duration, control, delivery format and arms were extracted from Procedure  2.The CES-D and LSI scores were extracted from Table 2. |
| Bryant, 2005 | 1.Culture was extracted from Participants; duration, control, delivery format and arms were extracted from Procedure  2.The raw data of pre-test mean and post-test mean of each group of Fordyce Happiness Score were obtained by contacting the author directly. |

| First Author & Year | Details of data extraction from included trials |
| --- | --- |
| Hurley, 2012 | 1.This study was published in a Dutch journal that is not indexed by PubMed. The full text is available through the PsycInfo database provided by the library.  2.Culture was extracted from Participants; duration, control, delivery format and arms were extracted from Procedure  2.The PANAS-X and BDI-II scores were extracted from Table 2. |
| Finan, 2023 | 1.Culture was extracted from Participants; duration, control, delivery format and arms were extracted from Interventions  2.The PANAS scores were extracted from Table 3. |
| Gold, 2023 | 1.Culture, duration, control, delivery format and arms were extracted from Methods  2.The PANAS-SF, PHQ and SAT scores were extracted from supplemental materials Table 1. |
| Mian, 2023 | 1.Culture, duration, control, delivery format and arms were extracted from Method  2.The PANAS scores were extracted from Table 7. |
| Fuju, 2023 | 1.Culture was extracted from Participants; duration, control, delivery format and arms were extracted from Procedure  2.The CES-D and WHO-5 scores were extracted from Table 3. |
| Spillane 2023 | 1.Culture, duration, control, delivery format and arms were extracted from Procedures  2.The PANAS-X scores were extracted from Table 2. |
| Bastiaansen, 2022 | 1.Culture was extracted from Study design; duration was extracted from Intervention; control, delivery format was extracted from Self-assessments; arm  was extracted from Participants  2.The IDS-SR scores were extracted from Supplemental materials Table 1b. |

*Note. HADS: Hospital Anxiety and Depression Scale; GDS: Geriatric Depression Scale; LSI: Life Satisfaction Index; MQOL-HK: McQill Quality of Life Index—Hong Kong version; Cochrane Handbook: Deeks et al., 2021; Higgins et al., 2021a; Higgins et al., 2021b; PANAS-X: Positive and Negative Affect Schedule Expanded Form: Joviality Scale; BDI-II: Beck Depression Inventory II; PSWQ: Penn State Worry Questionnaire; PANAS-PA: Positive and Negative Affect Scale – Positive Affect; CES-D: Center for Epidemiologic Studies Depression Scale; PHQ-9: Patient Health Questionnaire-9; TGT: Three Good Thing; SHS: Subjective Happiness Scale; GAD: General Anxiety Disorder Scale; LSIA: Life Satisfaction Index A; LSI: Life Satisfaction Index; PANAS-SF: Positive and Negative Affect Schedule-Short Form; SAT: Satisfaction with Life Questionnaire; WHO-5:World Health Organization‐Five Well‐Being Index; IDS-SR: Inventory for Depressive Symptomatology-Self-Report.*
